# Study of Position, Shape, and Size of Mental Foramen Utilizing Various Parameters in Dry Adult Human Mandibles from North India

**DOI:** 10.5402/2013/961429

**Published:** 2012-12-17

**Authors:** Virendra Budhiraja, Rakhi Rastogi, Rekha Lalwani, Prabhat Goel, Subhash Chandra Bose

**Affiliations:** ^1^Department of Anatomy, L.N. Medical College and J.K. Hospital, Sector-C, Sarvadhram, Kolar Road, Bhopal 460246, India; ^2^Department of Anatomy, L.L.R.M. Medical College, Meerut, India; ^3^Department of Anatomy, Subharti Medical College, Meerut, India; ^4^Department of Biochemistry, L.N. Medical College, Bhopal, India

## Abstract

*Background*. As the mental foramen (MF) is an important landmark to facilitate surgical, local anesthetic, and other invasive procedures, the present study was aimed to elucidate its morphological features and morphometric parameters with reference to surrounding landmarks. *Material and Method*. 105 dry adult human mandibles of unknown sex were observed for position, shape, and number of mental foramina. Their size was measured using a digital vernier caliper and statistically analyzed by mean and standard deviations (SD). *Results*. In most cases (74.3%), the MF was oval in shape and situated on the longitudinal axis of the 2nd premolar tooth (61% on right side and 59.1% on left side). The mean distance for the right and left sides was measured from various landmarks. *Conclusion*. Prior knowledge of mental foramen variations helps surgeons in planning surgery in that region to avoid nerve damage and also enable effective mental nerve block anesthesia.

## 1. Introduction

The mental foramen (MF) is situated on the anterolateral aspect of the body of mandible. It gives path to mental nerve and vessels [1–3]. Variations of the mental foramen are often encountered ranging from difference in shape and positions [4–6] to presence of accessory foramen [7] or even complete absence in some cases [8, 9]. Knowledge of its position, shape, and size is important for performing anesthetic block prior to clinical procedures in lower anterior teeth and to preserve integrity of the mental nerve trunk in surgical interventions [10, 11]. As the mental foramen is an important anatomical landmark to facilitate surgical, local anesthetic, and other invasive procedures, the present study is aimed at assessing morphological and morphometric features of mental foramen with reference to surrounding landmarks.

## 2. Material and Method

**Figure 1:**
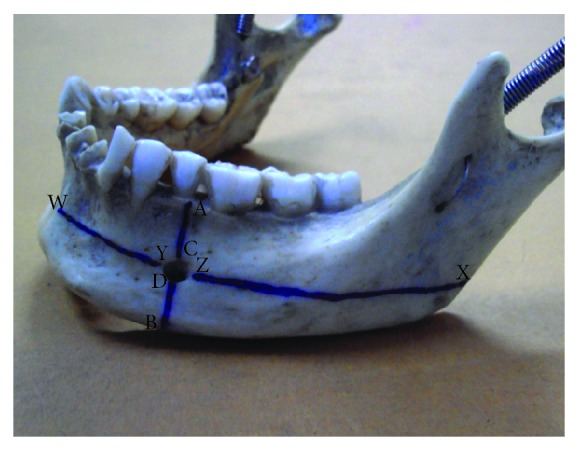
Relation of mental foramen to the body of mandible. A: point at alveolar crest lying on a longitudinal axis with mental foramen; B: point at lower border of mandible lying on a longitudinal axis with mental foramen; C: point at upper margin of mental foramen; D: point at lower margin of mental foramen; W: point at symphysis menti lying on a transverse axis with mental foramen; X: point at posterior border of ramus lying on a transverse axis with mental foramen; Y: point at medial margin of mental foramen; Z: point at lateral margin of mental foramen.

105 dry adult human mandible of unknown sex obtained from the Anatomy Department of L.L.R.M. and Subharti Medical College formed the material for study. We observed the position, shape, and number of MF. We measured the distance of MF (in mm) from various landmarks including symphysis menti, alveolar crest, posterior border of the ramus of mandible, and lower border of mandible with digital vernier caliper and calculated the size of mental foramen [2] (Figure 1).

AC: distance from alveolar crest to upper margin of mental foramen.
BD: distance from lower border of mandible to lower margin of mental foramen.
AB: distance from alveolar crest to lower border of mandible.
VD: vertical diameter of foramen  =  *Α*B − (*Α*C + BD).
WY: distance from symphysis menti to medial margin of mental foramen.
XZ: distance from posterior border of ramus of mandible to lateral margin of mental foramen.
WX: distance from symphysis menti to posterior border of ramus of mandible.
HD: horizontal diameter of foramen  =  WX − (WY + XZ).

The results are expressed as mean and standard deviations (SD).

## 3. Results

**Figure 2:**
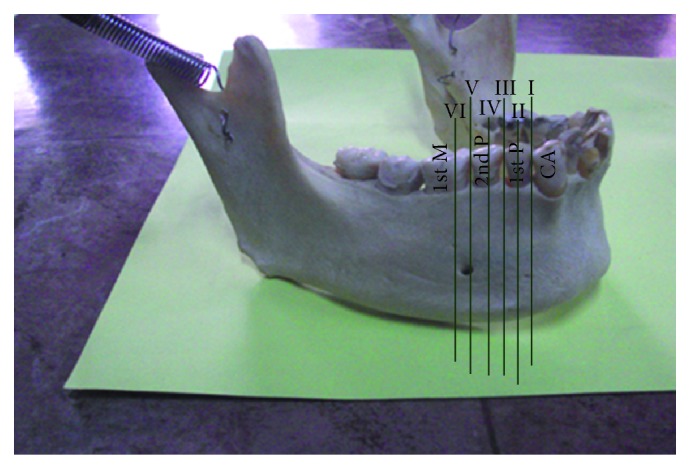
Variable relations of mental foramen to lower teeth as positions I–VI. CA: canine; 1st P: first premolar; 2nd P: second premolar; 1st M: first molar.

The position of MF was classified in relation to teeth of the lower jaw in accordance with Tebo and Telford [14] (Figure 2).

- Foramen lying on a longitudinal axis passing between canine and first premolar;
- foramen lying on the longitudinal axis of first premolar;
- foramen lying on a longitudinal axis passing between first and second premolars;
- foramen lying on longitudinal axis of second premolar;
- foramen lying on a longitudinal axis passing between second premolar and first molar;
- foramen lying on longitudinal axis of first molar.

**Figure 3:**
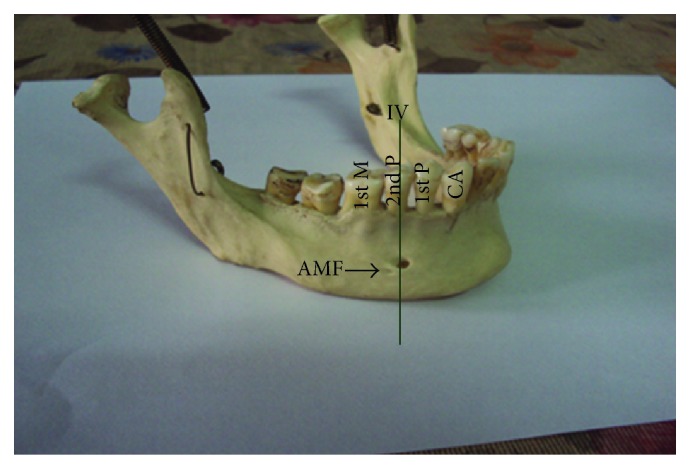
Right side of mandible showing mental foramen lying in position IV. CA: canine; 1st P: first premolar; 2nd P: second premolar; 1st M: first molar; AMF: accessory mental foramen.

**Figure 4:**
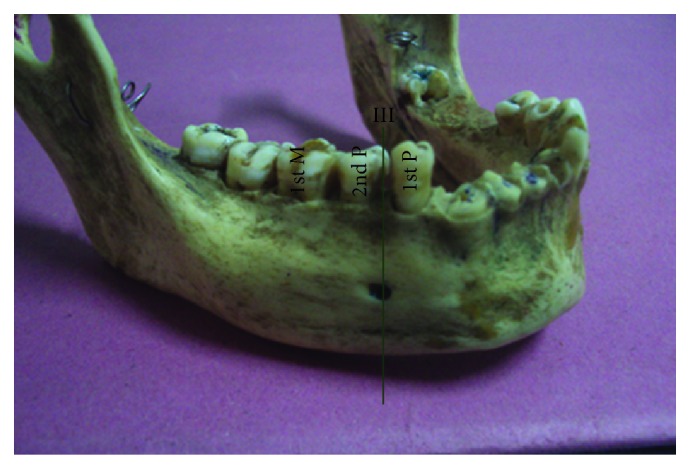
Right side of mandible showing mental foramen lying in position III. 1st P: first premolar; 2nd P: second premolar; 1st M: first molar.

**Figure 5:**
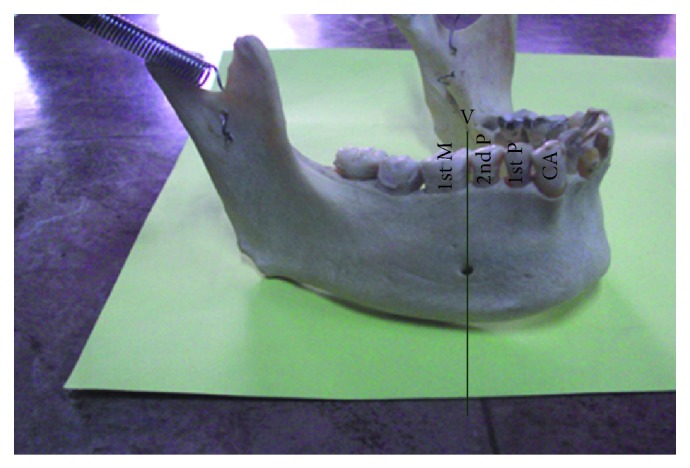
Right side of mandible showing mental foramen lying in position V. CA: canine; 1st P: first premolar; 2nd P: second premolar; 1st M: first molar.

**Figure 6:**
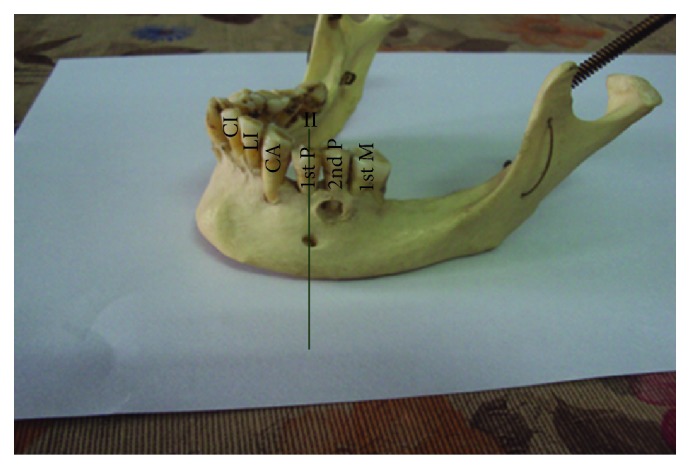
Left side of mandible showing mental foramen lying in position II. CI: central incisor; LI: lateral incisor; CA: canine; 1st P: first premolar; 2nd P: second premolar; 1st M: first molar.

**Figure 7:**
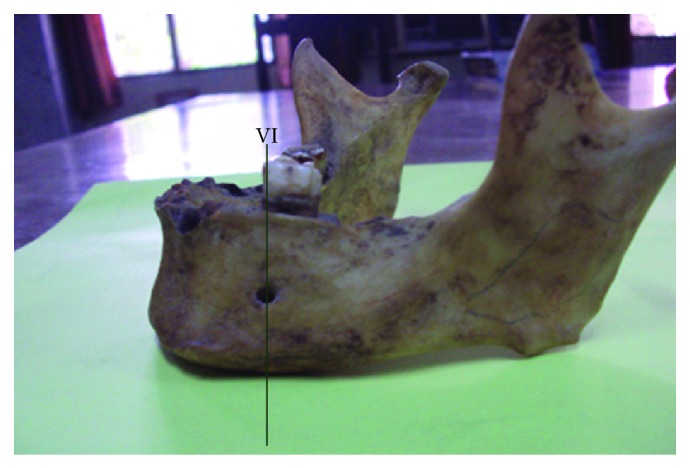
Left side of mandible showing mental foramen lying in position VI.

The most common position was on the longitudinal axis of second premolar (position IV) (Figure 3) followed by positions III (Figure 4), V (Figure 5), II (Figure 6), and VI (Figure 7). The MF was not observed in position I in any mandible. Results are presented in Table 1. To locate the MF and to measure its size, various parameters were considered and the results are presented in Tables 2 and 3.

In the majority of cases 78/105 (74.3%), the MF was oval in shape and in remaining cases 27/105 (25.7%) the mental foramen was round on the right side as well as on the left side. An accessory mental foramen (AMF) (Figure 3) was observed in 7 cases and was unilateral.

## 4. Discussion

The location of the MF is an important factor when considering the mental incisive anesthetic block and surgery in the outer premolar mandibular region. There are significant differences reported in the location of MF among different ethnic groups. Igbigbi and Lebona [2] in Malawians and Mbajiorgu et al. [15] in Zimbabweans mandibles reported position IV as the commonest followed by position V; however, Santini and Land [5] in British and Green [19] in Chinese mandibles observed position III being the commonest followed by position IV. In other studies on Kenyans mandibles [10], the position III was found most common followed by position II and in Malay populations [20] the most common position was IV followed by III, but in the above-mentioned studies right and left sides not considered separate from each other. In the present study, we considered right and left sides separately and compared the results with those of similar studies conducted in different population groups (Table 4) [4, 12, 13].

Variability in MF position may be related to different feeding habits subsequently affecting mandibular development [13]. Prior knowledge of common positions in local populations may be helpful in effective nerve blocks and surgeries in those regions. Considering the mean and respective SD of various parameters of MF in adult mandibles, the results of present study were similar to those observed by Amorim et al. [12] in Brazilian's mandible and Agarwal and Gupta [4] in mandibles from central India, but the mean of various measurements in Turkish [13] and Korean [21] mandibles were much lower than the present study. The differences observed among studies may be related to different methodology, such as measurements on skull photographs or use of different skull marks—center versus anterior or inferior margin of mental foramen or absence of skull mark information [11, 21].

We also measured the size of mental foramen. The mean VD of mental foramen in our study was 2.61 mm ± 0.17 mm on the right side and 2.53 mm±  0.14 mm on the left side, respectively and mean HD was 5.19 mm ± 0.24 mm on the right side and 5.12 mm±  0.28 mm on the left side, respectively. The results have been very close to those of Igbigbi and Lebona [2]; however, Oguz and Bozkir [22] did measurements in 34 dry mandibles of people from Turkey and found a mean HD of 2.93 mm on the right side and 3.14 mm on the left side, and a mean VD of 2.38 mm and 2.64 mm on the right and left sides, respectively. The present results differ significantly for HD from those of Oguz and Bozkir [22]. In another study conducted by Singh and Srivastav [16], only the HD was taken and the results showed the mean HD to be 2.79 mm on the right side and 2.57 mm on the left side, again much less than the present study. The probable reason for a significant difference in HD in their study was a higher number of rounds than oval MF. In the present study, we observed an oval-shaped MF in 74.3% mandibles and a round-shaped MF in 25.7%. A comparison between the results of the present study and previous ones is presented in Table 5 [4, 15–18].

The incidence of AMF varies in the literature. Singh and Srivastav [16] observed AMF in 13% mandibles; Gershenson et al. [17] examined 525 dry mandibles and reported that 4.3% mandibles had a double mental foramen and 0.7% mandibles had triple mental foramen; however, Serman [23] reported the incidence of AMF to be 2.7%. In the present study, we observed an AMF in 7/105 (6.6%) mandibles. An AMF is due to branching of mental nerve prior to its passing through mental foramen. Thus, the verification of the existence of an AMF would prevent nerve injury during periapical surgery.

## 5. Conclusion

Paralysis of the mental nerve is one of the principal complications of surgery of the mandibular canal and mental foramen regions. Therefore, identification of mental foramen in its various positions and its morphometric analysis is important for dental surgeons in nerve block and surgical procedures like apical curettage of mandibular premolars and periodontal surgery, to avoid injury to neurovascular bundle. In a majority of mandibles, we have found oval-shaped foramina lying in position IV. However, variations do exist in the position, shape, and size of mental foramen in different population groups. It is essential to be aware of the possibility of these anatomical variations while planning surgery in that region to avoid nerve damage and also to enable effective mental nerve block anesthesia.

## Figures and Tables

**Table 1:** The relation of the mental foramen to lower teeth *n* = 105.

Position	I	II	III	IV	V	VI
Right	0	4 (3.8%)	21 (20.0%)	64 (61.0%)	15 (14.3%)	1 (0.9%)
Left	0	3 (2.9%)	22 (20.9%)	62 (59.1%)	16 (15.2%)	2 (1.9%)

**Table 2:** Parameters to locate the right and left mental foramen in relation to lower border of mandible and alveolar crest, and foramen vertical diameter.

Parameters∗	AC		BD		AB		VD	
	Rt	Lt	Rt	Lt	Rt	Lt	Rt	Lt
Mean (mm)	11.46	11.33	15.25	15.40	29.30	29.20	2.61	2.53
SD (mm)	0.25	0.17	0.24	0.22	0.36	0.31	0.17	0.14

^*^AC: alveolar crest to upper margin of foramen; BD: lower border of mandible to lower margin of foramen; AB: alveolar crest to lower border of mandible; VD: vertical diameter of foramen.

**Table 3:** Parameters to locate the right and left mental foramen in relation to symphysis menti and posterior border of ramus of mandible, and foramen horizontal diameter.

Parameters∗	WY		XZ		WX		HD	
	Rt	Lt	Rt	Lt	Rt	Lt	Rt	Lt
Mean (mm)	25.39	25.29	65.76	66.13	96.34	96.57	5.19	5.12
SD (mm)	0.66	0.30	0.70	0.77	0.69	0.83	0.24	0.28

∗WY: symphysis menti to medial margin of foramen; XZ: posterior border of ramus of mandible to lateral margin of foramen; WX: symphysis menti to posterior border of ramus of mandible; HD: horizontal diameter of foramen.

**Table 4:** Comparison of present study with similar studies by different groups.

Authors	Population	Side	Positions of foramen in percent					
			I	II	III	IV	V	VI
Agarwal and Gupta [4]	Central Indian	Rt	0	0	7.8	81.5	2.7	7.9
		Lt	0	0	7.6	81.5	3.1	7.8
Amorim et al. [12]	Brazilian	Rt	0	0	19.8	71.4	8.8	0
		Lt	0	0	23.1	68.1	8.8	0
Yeşilyurt et al. [13]	Turkish	Rt	0	5.7	34.3	55.7	4.3	0
		Lt	0	7.1	25.7	61.4	5.7	0
Present study	Northern Indian	Rt	0	3.8	20.0	61.0	14.3	0.9
		Lt	0	2.9	20.9	59.1	15.2	1.9

**Table 5:** Comparison of shape of mental foramen between the present study and other studies.

Authors	Shape of mental foramen in percent	
	Oval	Round
Agarwal and Gupta [4]	92.0	8.0
Mbajiorgu et al. [15]	56.3	43.8
Singh and Srivastav [16]	6.0	94.0
Gershenson et al. [17]	65.5	34.5
Prabodra and Nanayakkara [18]	66.7	33.3
Present study	74.3	25.7
